# Imaging Dose, Cancer Risk and Cost Analysis in Image-guided Radiotherapy of Cancers

**DOI:** 10.1038/s41598-018-28431-9

**Published:** 2018-07-04

**Authors:** Li Zhou, Sen Bai, Yibao Zhang, Xin Ming, Ying Zhang, Jun Deng

**Affiliations:** 1 Division of Radiation Physics, State Key Laboratory of Biotherapy and Cancer Center, West China Hospital, Sichuan University, No. 37 Guo Xue Xiang, Chengdu, 610041 China; 2 Department of Radiation Oncology, Beijing Cancer Hospital, No. 52 Fucheng Road, Haidian District, Beijing, 100142 China; 3 Department of Biomedical Engineering, Tianjin University, No. 92 Weijin Road, Nankai District, Tianjin, 300072 China; 4 Department of Therapeutic Radiology, Yale University, 15 York Street, LL 508-Smilow, New Haven, Connecticut 06510-3221 USA

## Abstract

The purpose of this retrospective study is to evaluate the cumulative imaging doses, the associated cancer risk and the cost related to the various radiological imaging procedures in image-guided radiotherapy of cancers. Correlations between patients’ size and Monte Carlo simulated organ doses were established and validated for various imaging procedures, and then used for patient-specific organ dose estimation of 4,832 cancer patients. The associated cancer risk was estimated with published models and the cost was calculated based on the standard billing codes. The average (range) cumulative imaging doses to the brain, lungs and red bone marrow were 38.0 (0.5–177.3), 18.8 (0.4–246.5), and 49.1 (0.4–274.4) cGy, respectively. The associated average (range) lifetime attributable risk of cancer incidence per 100,000 persons was 78 (0–2798), 271 (1–8948), and 510 (0–4487) for brain cancer, lung cancer and leukemia, respectively. The median (range) imaging cost was $5256 (4268–15896) for the head scans, $5180 (4268–16274) for the thorax scans, and $7080 (4268–15288) for the pelvic scans, respectively. The image-guidance procedures and the accumulated imaging doses should be incorporated into clinical decision-making to personalize radiotherapy for individual patients.

## Introduction

Image-guidance has been widely used in the radiotherapeutic management of cancers, benefitting patients with significant margin reduction and normal tissue sparing. In addition, highly precise tumor targeting could lead to less geometrical miss and consequently improved therapeutic effects^1^. The most often applied image-guidance technologies nowadays include kilovoltage portal imaging (kVPI), megavoltage portal imaging (MVPI) and kilovoltage cone-beam computed tomography (kVCBCT), all of which involve ionizing radiation^2^. Yet, the cumulative imaging doses from various imaging procedures in image-guided radiotherapy (IGRT) have not been routinely considered in clinical workflow, due primarily to the absence of an efficient dose estimation method for individual patient.

Recent studies indicated that repetitive imaging scans can deposit considerable radiation doses to some radiosensitive organs (e.g., heart) and could cause higher radiogenic cancer risks to the patients undergoing IGRT, especially children^3,4^. Three recent epidemiological studies carried out in UK, France, USA and Australia have further confirmed the positive correlation between ionizing radiation and cancer risk in both children and radiation-monitored workers, reporting mean carcinogenic dose as low as 16 mGy^5,6^. With 14.1 million new cancer cases reported each year worldwide and the widespread applications of image guidance in cancer radiotherapy^7^, the increasing imaging doses in the IGRT patients may become a serious issue in the future.

Although there have been a lot of studies on imaging doses in radiotherapy using both Monte Carlo simulations and measurements^3,4,8–15^, a systematic investigation on the cumulative imaging doses from various radiological imaging procedures used in IGRT based on a large cohort has been missing. Therefore, the goal of this study is to retrospectively evaluate the cumulative imaging doses to the brain, lungs, and red bone marrow (RBM), the typical organs-at-risk (OARs) in the head, thorax and pelvis regions of all cancer patients who underwent IGRT at our institution in the past four-and-half years. Furthermore, the models proposed in the Biological Effects of Ionizing Radiation VII report (BEIR VII) were used to estimate the corresponding cancer incidence associated with the cumulative imaging doses^16^. The standard billing codes from the Centers for Medicare & Medicaid Services (CMS) of US government were used to calculate the cost related to various imaging procedures. In this work, all the patients undergoing radiotherapy received one or few of the 4 imaging procedures available in our institution including CT, kVCBCT, kVPI and MVPI. As there have been no MV-CT or Tomotherapy installed in our institution, we did not include the analysis of the cumulative imaging dose, second cancer risk and imaging cost from MV-CT scans or Tomotherapy in this paper.

## Methods

### Patient characteristics and data collection

All the cancer patients treated with radiotherapy at Yale-New Haven Hospital from September 1, 2009 to April 30, 2014 were reviewed with institutional review board (IRB) approval by Yale University Human Investigation Committee (HIC#1403013576). By the time when this retrospective study was initiated, all the reviewed patients who underwent radiotherapy at our institution have signed informed consent, finished their radiation treatments, and left our clinic already. Hence no separate informed consent was necessary. Patients treated without any image-guidance or computed tomography (CT) scans, and patients treated with total body irradiation or with large blocks protecting the brain, lungs or RBM, were excluded from this study. A total of 4,832 patients whose brain, lungs or RBM were irradiated by at least one of the three image-guidance modalities (i.e., kVCBCT, kVPI, MVPI) were included and all data were anonymized in this retrospective study. The characteristics of 4,832 patients were summarized in Table 1.

**Table 1:** Characteristics of 4832 patients in this study.

Characteristics	Patient (Organ)
Gender	
Male	2340
Female	2492
Age (years) at initial treatment	
Mean for Males	64
Range for Males	0–96
Mean for Females	61
Range for Females	1–99
Year of initial treatment	
2009	202
2010	938
2011	1056
2012	1117
2013	1165
2014	354
Organs irradiated by imaging	
Brain only	1036
Lungs only	2303
Red bone marrow only	1015
Brain and lungs (no red bone marrow)	194
Brain and red bone marrow (no lungs)	49
Lungs and red bone marrow (no brain)	161
Brain, lungs and red bone marrow	74

The gender, age, numbers and types of image-guidance procedures performed on these 4,832 patients were obtained directly from ARIA record & verify system (Varian Medical Systems, Palo Alto, CA). One axial CT slice at the eyebrows, nipples, or hip level was exported from Eclipse treatment planning system via DICOM, based on which the circumference of the head, thorax or pelvis was computed using the DICOMan software^17^. The patients’ circumferences (surrogates of patients’ size) ranged from 39 to 66 cm, 42 to 160 cm, and 37 to 168 cm in the head, thorax and pelvis regions, respectively.

### Monte Carlo simulations of CT, kVCBCT, kVPI and MVPI

An EGSnrc/BEAMnrc code was used to simulate the photon beams emanated from the X-ray sources of CT, kVPI, MVPI and kVCBCT systems, respectively^18,19^. Particularly, the default settings such as kVp, mAs, blades and bowtie filter specific to individual scan protocol were used. The phase space data were first scored below the last beam-shaping device (e.g., bowtie filter in kVCBCT) and then used to derive the multiple source model for the corresponding scan protocol^20,21^.

MCSIM, an EGS4/BEAM user code, was used to calculate the three-dimensional dose distributions in patient anatomy with multiple source model as beam input for each imaging procedure^22,23^. To convert Monte Carlo simulations into absolute dose, the absorbed dose was first measured at the isocenter of an acrylic ball phantom of 5 cm in diameter with a calibrated EXRADIN A12 ionization chamber (Standard Imaging, Middleton, WI) for the specific scan protocol^24^. The EXRADIN A12 ionization chamber was calibrated every two years by an Accredited Dosimetry Calibration Laboratory (ADCL) traceable to the National Institute of Standards and Technology (NIST). In performing the in-phantom ion chamber measurement, the ratio of mass energy-absorption coefficient for water to air and other beam-quality and chamber-related correction factors were applied per TG-61 protocol^24^. Monte Carlo simulation was then performed to a chamber volume inside the ball phantom with the same beam setup using multiple source model. The ratio of the measured dose to the Monte Carlo simulation yielded a conversion factor, which was unique to the clinical setup and beam configuration. We repeated the process for all the imaging devices and protocols used in our clinic and obtained corresponding conversion factors. With all those conversion factors, all the Monte Carlo simulations in patient CT anatomy can be converted into absolute doses to water.

The following imaging devices and protocols were routinely used in our clinic and modeled with Monte Carlo simulations:

1. A GE LightSpeed 16-slice CT scanner: head protocol (helical scan, 120 kV, 304 mAs, pitch 0.938); chest protocol (axial scan, 140 kV, 250 mAs); and pelvis protocol (axial scan, 140 kV, 250 mAs).
2. OBI kVCBCT systems mounted on Varian accelerators: high-quality head protocol (100 kV, 720 mAs, 204° arc, full bowtie); low-dose thorax protocol (110 kV, 262 mAs, 364° arc, half bowtie); and pelvis protocol (125 kV, 680 mAs, 364° arc, half bowtie).
3. Paired kVPI on Varian OBI systems: 180° and 270° gantry, 120 kV, 126 mAs, no bowtie filter, average field X1 = X2 = 13.3 cm, Y1 = Y2 = 10.0 cm.
4. Paired MVPI on Varian accelerators: 6-MV, 2 MU, 180° and 270° gantry, average field = 20 cm × 20 cm for the head, 22.8 cm × 20.1 cm for the chest, and 21.5 cm × 20.1 cm for the pelvis respectively.

In all the Monte Carlo simulations, the energy cutoff for electrons (ECUT) = the threshold for δ-ray (AE) = 521 keV, and the energy cutoff for photons (PCUT) = the threshold for bremsstrahlung (AP) = 10 keV. A statistical uncertainty (1σ) of 2% has been achieved for all the Monte Carlo simulations in this study.

### Organ dose and lifetime attributable risk estimation

Using SigmaPlot suite, Monte Carlo-simulated doses to the brain, lungs and RBM deposited by CT, kVPI, MVPI and kVCBCT scans were regressed as empirical functions of the corresponding circumference, respectively. Using the established empirical functions, mean organ doses of 4,832 patients from 142,824 imaging procedures were estimated and accumulated. The lifetime attributable risk (LAR) of cancer incidence based on BEIR VII models^16^ was calculated to quantify the probability of cancer incidence associated with the cumulative imaging doses in IGRT.

The LAR for a person exposed to dose D (Sv) at age e (years) was defined as

1 $${\rm{LAR}}({\rm{D}},{\rm{e}})=\sum _{a}^{100}{\rm{M}}({\rm{D}},{\rm{e}},{\rm{a}})\cdot \frac{S(a)}{S(e)}$$

where a denotes attained age (years), M(D, e, a) is the excess absolute risk (EAR), S(a) denotes the probability of surviving until age a, and S(a)/S(e) denotes the probability of surviving to age a conditional on survival to age e.

For brain and lung cancer,

2 $${\rm{ERR}}\,{\rm{or}}\,{\rm{EAR}}({\rm{D}},{\rm{s}},{\rm{e}},{\rm{a}})={{\rm{\beta }}}_{{\rm{s}}}{\rm{D}}\,\exp ({{\rm{\gamma }}{\rm{e}}}^{\ast }){(\frac{a}{60})}^{{\rm{\eta }}}$$

where ERR stands for the excess relative risk.

For leukemia,

3 $${\rm{ERR}}\,{\rm{or}}\,{\rm{EAR}}({\rm{D}},{\rm{s}},{\rm{e}},{\rm{t}})={{\rm{\beta }}}_{{\rm{s}}}{\rm{D}}(1+{\rm{\theta }}{\rm{D}})\exp [{{\rm{\gamma }}{\rm{e}}}^{\ast }+{\rm{\delta }}\,\mathrm{log}(\frac{{\rm{t}}}{25})+{\rm{\varphi }}{{\rm{e}}}^{\ast }\,\mathrm{log}(\frac{{\rm{t}}}{25})]$$

All the parameters in the equations such as e*, θ, ϒ, η, δ, and φ were extracted from BEIR VII report. An in-house MATLAB code was developed for site-sp ecific LAR estimates.

### Imaging cost analysis

CMS standard billing codes (applicable to USA only) were used in this study to calculate the imaging cost associated with the various imaging procedures performed on the 4,832 patients. Specifically, $3,828 was charged for each CT scan. $76, $76 and $118 were charged to each MVPI, kVPI and kVCBCT procedure, respectively, on top of a one-time fee of $440. The proportions of kVCBCT, MVPI and kVPI used in each lesion site for all the 4,832 patients were calculated and the summation of all the cost related to various procedures yielded the total imaging cost for each patient.

### Statistical analysis

Descriptive Statistics and Mann-Whitney Rank Sum Test were conducted using SigmaPlot suite (Version 12.0, Systat Software, San Jose, CA). A P-value less than or equal to 0.05 indicates a significant difference.

## Results

### Validation of Monte Carlo simulations

**Figure 1:**
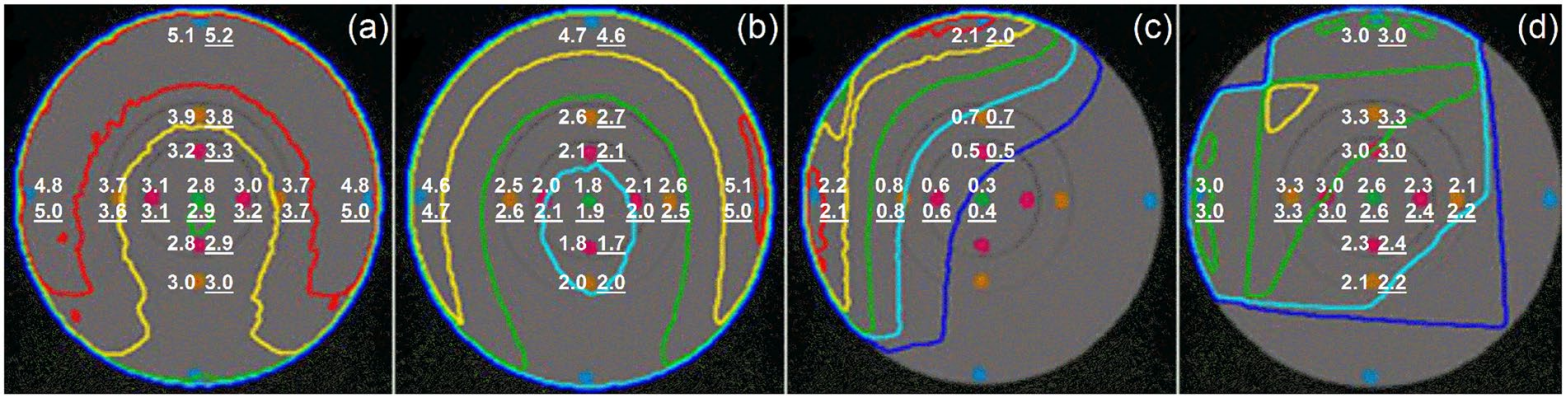
Comparison of absolute doses in a 32-cm diameter CTDI phantom between Monte Carlo simulations and measurements for (**a**) a pelvic CT scan, (**b**) a pelvic kVCBCT scan, (**c**) a default kVPI, and (**d**) a default MVPI. White values indicate the measured and simulated point doses. The measured values are underlined for the sake of distinction. Only isodose lines of 20, 40, 60, 80 and 100% are shown for clarity.

As shown in Fig. 1, Monte Carlo simulations of 4 imaging devices/protocols were compared with the ion chamber measurements in a 32-cm diameter CTDI phantom for (a) CT, (b) kVCBCT, (c) kVPI and (d) MVPI. The measured and simulated absolute doses (in cGy) were indicated with red and blue values for a variety of points located at the center, 3, 6, 9 and 12 o’clock positions, respectively. For clarity, only isodose lines of 20%, 40%, 60%, 80% and 100% of 4.5 cGy, 5.0 cGy, 2.0 cGy and 5.0 cGy were shown on (a) to (d), respectively. The relative differences of absolute dose between the Monte Carlo simulations and the ion chamber measurements ranged from 0.8% to 5.0% for CT, from 1.0% to 5.3% for kVCBCT, from 1.0% to 5.0% for kVPI, and from 0.3% to 2.9% for MVPI, respectively. Overall, the ion chamber measurements have confirmed the dose calculation accuracy of our Monte Carlo simulations of the imaging procedures to within 5.3%. Hence, these validated Monte Carlo models were employed in the subsequent population-based dose study in patient anatomy. Empirical functions were used to describe correlations between patient size and structural mean dose, whose parameters are listed in Table 2, where the empirical functions of kVCBCT for lungs and RBM were published in our previous work^4,8^.

**Table 2:** The empirical functions used to estimate the imaging doses to the brain, lungs and red bone marrow scanned by various imaging procedures, in which, C was the circumference of the head, thorax or pelvis, respectively.

	Imaging Dose (cGy) =	Fitting Parameters		
CT	y0 × exp(−a × C)	y0	a	
Brain		1.23	1.49E-02	
Lung		1.48	9.50E-03	
Red bone marrow		2.17	1.38E-02	
kVPI	y0 + a × C	y0	a	
Brain		2.0	−1.69E-02	
Lung		2.05	−1.15E-02	
Red bone marrow		1.95	−1.27E-02	
MVPI	y0 + a × C	y0	a	
Brain		4.32	−1.46E-02	
Lung		4.46	−1.32E-02	
Red bone marrow		4.07	−1.54E-02	
CBCT		y0	a	b
Brain	y0 + a × C + b × C^2^	29.02	−8.01E-01	0.0063
Lung	y0 + a × C	3.61	−2.12E-02	
Red bone marrow	y0 + a × lnC + b × Age	33.55	−6.21E + 00	−6.21

### Patterns of image-guidance procedures

**Figure 2:**
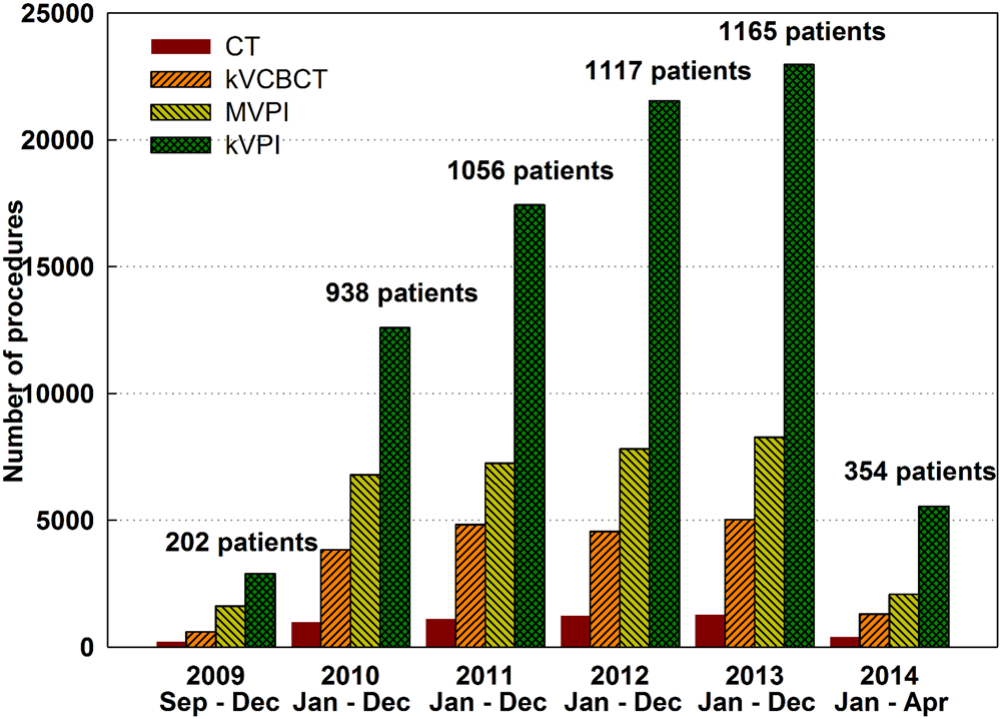
CT scans, image-guidance procedures and patients reviewed in this study.

Figure 2 shows the statistics of various imaging procedures and the new patients receiving IGRT during the past four-and-half years at our institution. Overall, there has been a steady rise in the numbers of new patients and imaging procedures each year from 2009 to 2013, followed by a decrease in 2014. Compared to the previous year, the total number of imaging procedures increased by 52.1%, 26.6%, 14.7%, and 6.8%, respectively from 2010 to 2013. The decrease of imaging procedures in 2014 was primarily due to the decrease of new patients treated in the first months of 2014. KVCBCT, MVPI and kVPI accounted for 14.1%, 24.1 and 58.1% of all the 142,824 imaging procedures performed on 4,832 patients, respectively. The average CT, kVCBCT, MVPI and kVPI scans per patient were 1.1, 4.2, 7.1 and 17.2, respectively.

### Cumulative imaging doses to the brain, lungs and RBM

**Figure 3:**
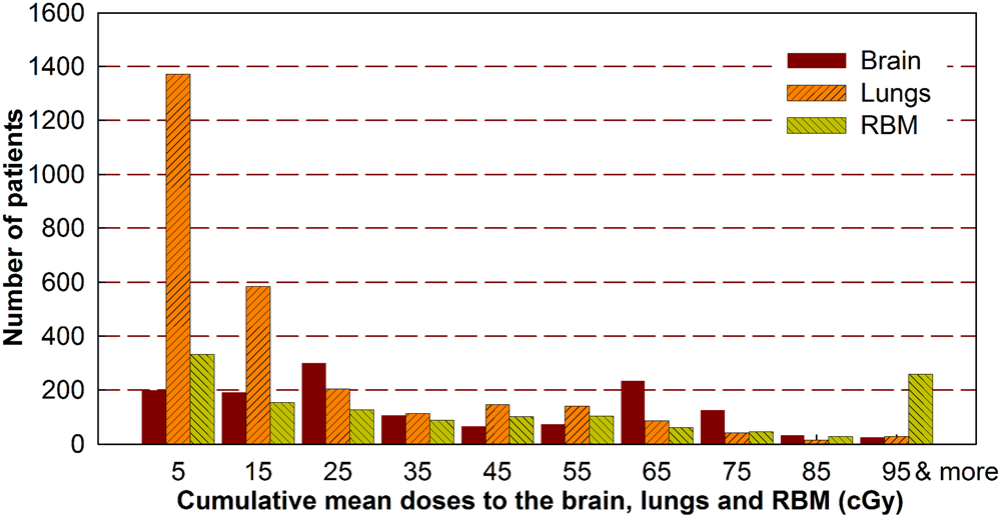
Cumulative imaging doses to the organs-at-risk from CT, MVPI, kVPI and kVCBCT combined.

As shown in Fig. 3, since different image-guidance procedures were used in the head, thorax and pelvis regions, the dose depositions to the regional OARs were quite different. The majority of our patients received 15 cGy or less imaging doses to the lungs, yet the imaging doses to the brain and RBM ranged from 5 to 75 cGy for most patients. Among 5,384 organs being irradiated, the average (range) cumulative imaging doses to the brain, lungs and RBM were 38.0 (0.5–177.3), 18.8 (0.4–246.5), and 49.1 (0.4–274.4) cGy, respectively. Out of 4,832 patients, 63.8%, 88.7% and 61.9% of them received 50 cGy or less doses to the brain, lungs and RBM, respectively. Yet, 272 organs (19 brain, 19 lungs and 234 RBM volumes), which accounted for 5% of patients in this study, received 100 cGy or more doses with the maximum doses of 177.3, 246.5 and 274.4 cGy, respectively. Among these 272 organs with high doses, 6 brain and 5 lung volumes were from the patients younger than 20 years old. These high doses were found to be largely caused by the repetitive imaging procedures and non-personalized scan settings.

### The LAR of cancer incidence

**Figure 4:**
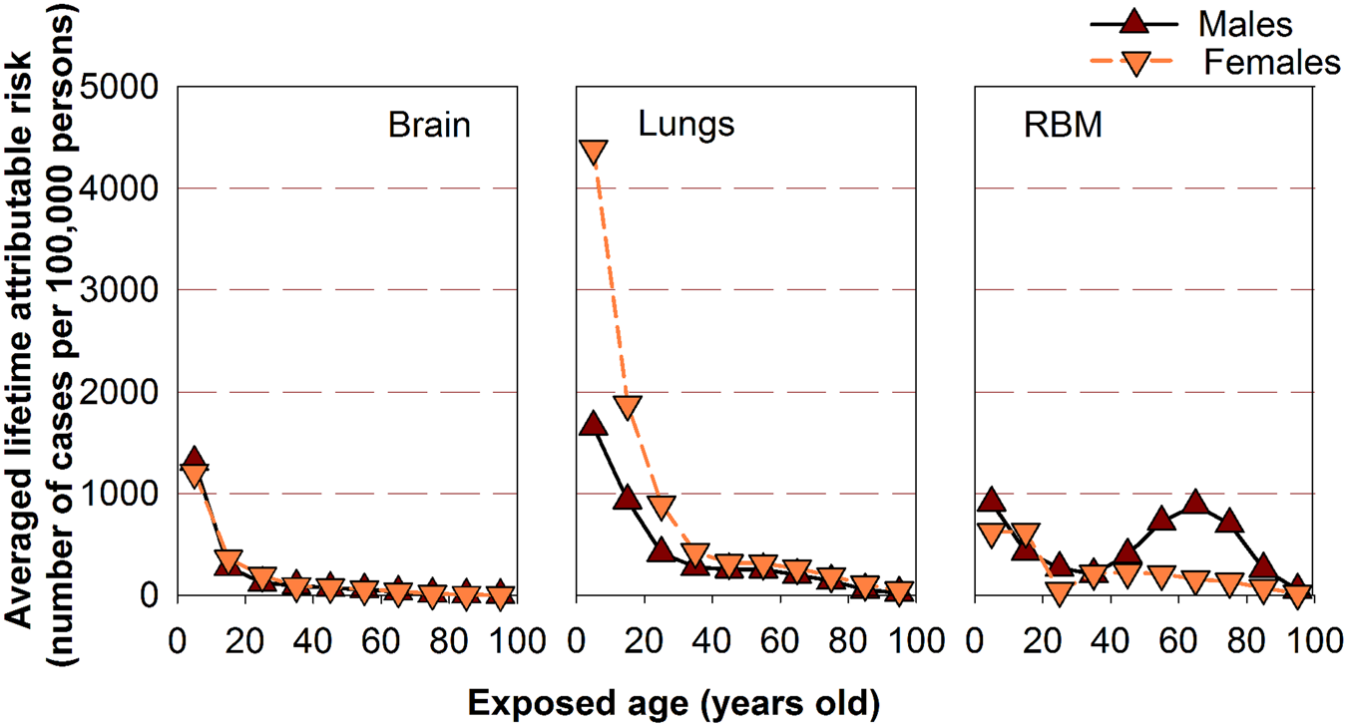
The averaged lifetime attributable risk of brain, lung cancers and leukemia as a result of imaging doses.

Figure 4 depicts for both males and females, the correlations between the exposed age and averaged LAR of cancer incidence as a result of cumulative imaging doses to the brain, lungs and RBM, respectively. For both genders, the averaged LAR of incidence for brain and lung cancers decreased monotonically with age. However, the LAR of leukemia incidence displayed an unusual trend with a regular decrease in young groups followed by a “hump” in senior groups. The hump peaks around 65 years old for males due largely to the frequent kVCBCT scans in prostate IGRT, whereas it peaks around 45 years old for females due to the increased image-guidance in radiation treatments of pelvic lesions. Regardless of age, a statistically significant difference was observed for the LAR of both lung cancer and leukemia incidence between the males and females (p < 0.001), but was not present in the LAR of brain cancer incidence (p = 0.063). The difference between females and males for lung cancer LAR was largely due to the larger β_S_ factor for the females in the BEIR VII model.

### Cost of imaging procedures in IGRT

**Figure 5:**
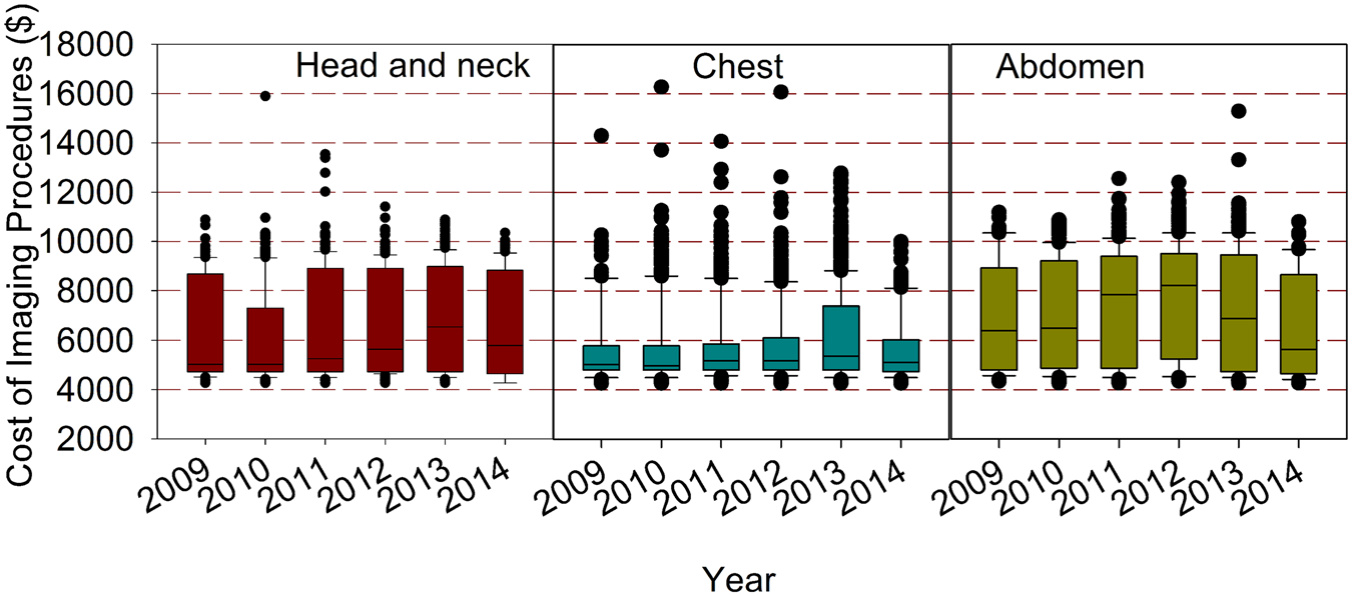
The distribution of total imaging cost per patient at three anatomic sites from 2009 to 2014.

Figure 5 shows the total cost of imaging procedures per patient for all the 4,832 patients from 2009 to 2014 with the 90^th^, 75^th^, median, 25^th^, and 10^th^ percentiles indicated in the box plots and the outliers shown as solid symbols. Generally speaking, the median imaging cost experienced a gradual rise followed by a gentle decrease for each lesion site from 2009 to 2014. Specifically, the median imaging cost from 2009 to 2014 was $5028, $5028, $5256, $5636, $6548, $5788 in the head, $5028, $4976, $5180, $5180, $5370 and $5104 in the chest, and $6396, $6510, $7840, $8220, $6890 and $5636 in the pelvis, respectively. In any given year, the differences in the medians among the three sites are statistically significant (p = 0.016 for 2009, p < 0.001 from 2010 to 2013, and p = 0.025 for 2014). The median of the total imaging cost per patient in IGRT from 2009 to 2014 was $5180, $5180, $5256, $5465, $5484 and $5330, respectively.

## Discussion

In this study, organ-specific correlations between imaging dose and patient size were first established for various imaging procedures based on Monte Carlo simulations in patient anatomy. Subsequently, the established empirical functions (Table 2) coded with MATLAB were used to estimate organ doses when the OARs were irradiated by various imaging procedures in IGRT. In general, 64.4% of our 4,832 patients received dose of more than 10 cGy (equivalent to 100 mSv) from imaging procedures, 85.2%, 49.3% and 74.4% of which are in the brain, lungs and RBM groups, respectively. In addition, our results indicated that the cumulative imaging doses may not be considered negligible for a certain group of patients undergoing IGRT. For example, for children younger than 15 years, 5–10 abdominal CT scans or 2–3 head CT scans will result in a cumulative imaging dose of 5 cGy to the RBM or 6 cGy to the brain, respectively^5^. In our study, among the 59 children younger than 15 years, the average cumulative imaging doses to the brains and the RBMs were 64.4 cGy and 46.0 cGy, respectively, with the associated LAR 10 and 8 times higher than that from the CT scans^5^.

The cumulative imaging dose depended on the frequency of imaging acquisitions and the radiation dose of each procedure: the latter was directly related to the patient size and the scan settings. For example, using the default settings of CT, kVCBCT, kVPI and MVPI, the mean doses to the lungs were 0.5, 1.1, 0.7 and 2.9 cGy for an adult with a chest circumference of 120 cm, but were 0.8, 2.3, 1.4 and 3.7 cGy for a child with a chest circumference of 60 cm, respectively. The excessive dose to the child from the default settings was clinically unjustifiable and could be largely avoided by personalized imaging protocols^25^.

In the image-guided radiotherapy of cancers, there are the therapeutic doses used to kill the cancerous tissues as well as the imaging doses used for tumor localization. The ratio of the imaging dose to the therapeutic dose depends on the patient size, the prescription dose, the imaging modality, the frequency and the settings of the applied image-guidance procedures. In the studied patients, it was found that the average ratio (range) of the imaging dose to the therapeutic dose was 0.65% (0.01–7.59%), with about 0.2% of patients having a ratio larger than 5%. The benefit/risk of image-guidance should be carefully evaluated for this small group of patients.

Recently there have been a series of studies on the scatter and leakage doses from linear accelerators and the associated secondary cancer risk^26–31^. Vu Bezin *et al*. reported that the leakage doses from 6 MV photons were similar to those delivered during CT scans (0.2–6 cGy for a 70 Gy delivery at isocenter), and the low doses should not be neglected while estimating the secondary cancer risk^30^. In our study, 36.2%, 11.3% and 38.1% of patients received cumulative imaging doses of 50 cGy or more to the brains, lungs and RBM, respectively. The secondary cancer risk from the imaging doses may be comparable to that from the leakage dose in conformal and intensity-modulated radiation therapy^26,27^. Besides this preliminary study, long-term follow-up and prospective clinical trials will be much needed to confirm what kind of effect the cumulative doses from various radiological imaging procedures may have on the patients undergoing IGRT particularly children.

The imaging cost for a cancer patient consists of a fixed charge and a variable charge. While the fixed charge has been standardized per CMS billing codes, the variable charge depends on both the frequency and the type of image-guidance procedures. An optimized choice of the frequency and the procedure type could help reduce the imaging cost while maintaining a high quality for radiation treatment. Based on this study, the average imaging cost per patient was $6197, $6183, $6358, $6428, $6535 and $6092 from 2009 to 2014, respectively. An optimized and personalized application of the image-guidance procedures for each patient would help deliver a cost-effective health care in the radiotherapeutic management of cancers^32,33^. For example, we can personalize scan range for the individual patient, restrict the use of fluoroscopy, or choose alternative imaging modalities such as magnetic resonance imaging and ultrasound. Also, we should apply not only site-specific but also size-specific protocols to minimize radiation doses while maintaining acceptable imaging quality^34^.

It is important to recognize the importance of image-guidance in cancer radiotherapy as well as its potential risk^1,35,36^. On one hand, with image-guidance, the significant shrinkage of CTV-PTV margin will reduce not only the volume of healthy tissues near target exposed to higher doses of radiation but also the volume of normal tissues distal from target exposed to lower doses, hence resulting in a decrease in second cancers. On the other hand, as a result of smaller margins and better positioning with IGRT, higher therapeutic doses are more frequently delivered with modern advanced radiotherapy techniques such as SRS, SBRT and VMAT, which may increase the risk for second cancers in those patients if the tumoricidal doses were not delivered as planned due to intra-fraction organ motion or deformed target volume.

While the effects of high and acute doses of ionizing radiation are easily observed and understood in humans such as Japanese Atomic Bomb survivors, the effects of low-level radiation are very difficult to observe and highly controversial. This is because the baseline cancer rate is already very high and the risk of developing cancer fluctuates significantly with individual life style and environmental factors, obscuring the subtle effects of low-level radiation. This is especially true for the patients undergoing image-guided radiotherapy where a large lethal dose in the order of tens of Gy is intended for the tumor killing while a small imaging dose in the order of cGy to tens of cGy is used for tumor localization and alignment. However, besides the confirmed positive correlation between ionizing radiation and cancer risk in both children and radiation-monitored workers^5,6^, Mathews *et al*. have reported a dose-response relation in 680,000 children and adolescents with increased incidence of cancer due to exposure to low dose diagnostic CT scans at 4.5 mSv per scan^37^. Rampinelli *et al*. have recently demonstrated that the median cumulative radiation exposure from low dose CT screening over 10 years was 9.3 mSv for men and 13.0 mSv for women, respectively, with an non-negligible but acceptable cancer risk^38^. All of these studies have taken many years to finish. Hence, we expect that it would take a long-term investigation with collaborative efforts to determine the association of cancer with the imaging doses for a large patient population who undergoes IGRT of cancers.

While most of procedures are clinically justified by the benefit outweighing the potential risk, we should be prudent about the application of image-guidance in a small portion of patients who may receive dangerously high doses to some critical organs as a result of non-personalized scan settings and over-imaging. One end product of this retrospective study was the creation of an institutional ‘Big Data’ repository consisting of patient gender, age, size, treatment history, imaging procedures, shifts as well as organ dose depositions. Moving forward, we plan to track the organ doses for all the patients, particularly those with imaging doses higher than 100 cGy, considering the imaging doses as well as the scatter and leakage doses from the mega-voltage radiation treatments. A comprehensive understanding of organ doses would help the clinicians tailor radiotherapy for each of their patients.

## Conclusion

In conclusion, our results suggest that it is essential to evaluate the cumulative imaging doses to personalize image-guidance and radiation treatment for individual patient undergoing IGRT. Appropriate usage of image-guidance procedures is highly desired to maintain a cost-effective health care.

### Data availability

The corresponding author has full access to all the data in the study and final responsibility for the decision to submit for publication. The data in this study will be available from the corresponding author upon request.
